# Attentional Rhythms Are Sensitive to Binocular Visual Pathway

**DOI:** 10.1002/pchj.826

**Published:** 2025-01-08

**Authors:** Bo Dong, Guangyao Zu, Ying Zou, Jianrong Jia, Airui Chen, Ming Zhang

**Affiliations:** ^1^ Department of Psychology Suzhou University of Science and Technology Suzhou China; ^2^ Department of Psychology Soochow University Suzhou China; ^3^ Institute of Psychological Sciences Hangzhou Normal University Hangzhou China; ^4^ Faculty of Interdisciplinary Science and Engineering in Health Systems Okayama University Okayama Japan

**Keywords:** attentional rhythms, binocular vision, impulse response function, V1

## Abstract

Visual attention is intrinsically rhythmic and oscillates based on the discrete sampling of either single or multiple objects. Recently, studies have found that the early visual cortex (V1/V2) modulates attentional rhythms. Both monocular and binocular cells are present in the early visual cortex, which acts as a transfer station for transformation of the monocular visual pathway into the binocular visual pathway. However, whether the neural site of attentional rhythms is in the monocular or binocular visual pathway needs further study. In the current study, we leveraged the anatomical features of the monocular and binocular pathway to design a paradigm with same‐eye and different‐eye presentations of cues and targets. By combining this approach with EEG recordings and analysis the impulse response function (TRF), we aimed to address this question. In Experiment 1, we reset the phase of attentional rhythms in one monocular channel (left eye or right eye) by a dichoptic cue and tracked the impulse response function (TRF) of the monocular channel in the left and right eye separately. We found no significant differences in the respective TRFs and their spectra for each eye, suggesting that attention rarely switched between the two eyes, indicating that the binocular visual pathway, not the monocular visual pathway, is the neural site of attentional rhythms. These results were verified when resetting the phases of attentional rhythms by a binocular cue in Experiment 2. These results suggest that attentional rhythms may be sensitive to activities in the binocular visual pathway.

## 1 Introduction

Recently, several studies found that sustained attention works in a rhythmic manner, not as a continuously active spotlight over time (Fiebelkorn, Saalmann, and Kastner 2013; Landau and Fries 2012; VanRullen 2016, 2018). Visual attention thus manifests itself as a rhythmic spotlight and divides visual information into a series of temporal chunks with a length of several hundred milliseconds (Busch and VanRullen 2010; Landau et al. 2015). This cyclical manifestation is called the attentional rhythms (Chen, Tang, et al. 2017; Chen, Wang, et al. 2017; Fiebelkorn and Kastner 2019; Herbst and Landau 2016; Jensen, Bonnefond, and VanRullen 2012; VanRullen 2016, 2018). Attentional rhythms modulate our perceptual performance and manifest themselves in various behavioral oscillations. When we deploy attention on a single location or multiple locations (i.e., location‐based attention), theta and alpha oscillations are found in aspects of processing efficiency, such as reaction time, accuracy, and sensitivity (d‐prime) (Chen, Tang, et al. 2017; Chen, Wang, et al. 2017; Chen et al. 2020; Dugué, Marque, and VanRullen 2015; Dugué et al. 2015; Landau and Fries 2012; Song et al. 2014; VanRullen, Reddy, and Koch 2005; Zhang, Morrone, and Alais 2019). Similarly, behavioral oscillations were found in object‐based attention (Fiebelkorn, Saalmann, and Kastner 2013) and feature‐based attention (Mo et al. 2019; Re et al. 2019). Interestingly, some top‐down and bottom‐up factors, such as the saliency of the target, the predictability of cues, task difficulty, task rewards, and cortical distance, could modulate the patterns of attentional rhythms (Chen, Tang, et al. 2017; Chen, Wang, et al. 2017; Chen et al. 2018, 2020; Dugué, Xue, and Carrasco 2017; Su et al. 2021). Previous studies employed a behavioral technique with high‐temporal‐resolution for detecting attentional rhythms (Chen, Tang, et al. 2017; Chen, Wang, et al. 2017; Chen et al. 2018, 2020; Landau and Fries 2012; Song et al. 2014). This technique becomes the primary means of studying the temporal characteristics of covert attention. The method uses a salient cue to reset attentional phase, followed by targets at various stimulus onset asynchronies (SOAs). SOAs are sampled at high temporal resolution (every 10–20 ms) to capture fine‐grained attentional changes. The key outcome is the dynamic change in detection performance over time, representing attentional dynamics. After preprocessing, a Fast Fourier Transform reveals the frequency‐domain information of behavioral oscillations, showing attention's rhythm frequency. This method has been widely applied in cue‐target and visual search paradigms, providing new insights into attention's rhythmic nature with unprecedented temporal precision.

Although attentional rhythms are robust and evident in different paradigms of studying visual attention, our knowledge regarding the neural site(s) of origin of this phenomenon is scarce. Previous studies suggested that the early visual cortex (V1/V2) may be involved in the production of attentional rhythms. The early visual cortex is the first neural site(s) of visual information processing in the cerebral cortex, and its role in visual attention has been confirmed by numerous studies (Hembrook‐Short et al. 2019; Klein et al. 2018; Motter 1993). Given the putative role of the occipital cortex in visual attentional oscillations, Dugué and colleagues employed transcranial magnetic stimulation (TMS) to modulate activity in V1/V2. They observed that when the TMS frequency approximated that of the behavioral oscillations (approximately 5.7 Hz), the most pronounced effects were seen in both visual search efficiency (Dugué, Marque, and VanRullen 2015; Dugué et al. 2015) and detection accuracy in cue‐target paradigms (Dugué, Roberts, and Carrasco 2016). These findings suggest that perturbation of V1/V2 activity significantly influences behavioral oscillations, thereby implicating these early visual areas in the generation or maintenance of attentional rhythms (Dugué and Van Rullen 2017). However, given the spatial imprecision of TMS interference, which affects areas 2–5 cm in radius even with high‐precision coils (Wassermann et al. 2008), and the structural and functional complexity of V1/V2, which encompasses both monocular and binocular visual pathways (Hubel and Wiesel 1977; Wandell 1995), the precise neural substrates and pathways underlying visual attention rhythms have not been fully understood.

Hubel, Wiesel, and colleagues' seminal studies (Hubel and Wiesel 1977; Livingstone and Hubel 1987) revealed that monocular information remains segregated through the precortical pathway up to layer IVc of V1, termed the monocular pathway. In V1, monocular cells, responsive only to same‐eye stimulation and preserving eye‐of‐origin information, form ocular‐dominance columns. Binocular convergence starts in V1's layer IVc, with subsequent layers (e.g., II and III) and higher areas (V2, V3) containing binocular cells responsive to either eye's input, thus losing eye‐of‐origin specificity. This anatomical organization enables researchers to localize neural sites of visual phenomena: effects differing between same‐eye and different‐eye conditions implicate the monocular pathway (pre‐IVc), while effects insensitive to eye of origin suggest involvement of binocular circuits (IVc and beyond, including higher cortical areas like V2, V4, MT/V5, or LIP). The logic of comparing behavioral or neural performances between same‐eye and different‐eye conditions has been widely applied to investigate neural sites of various visual phenomena, including orientation adaptation (Gilinsky and Doherty 1969), spatial frequency adaptation (Blakemore and Campbell 1969), color adaptation (McCollough 1965), motion aftereffect (Anstis, Verstraten, and Mather 1998), subjective contour perception (Paradiso, Shimojo, and Nakayama 1989), perceptual learning (Schoups and Orban 1996), and attention saliency map (Zhaoping 2008).

The unique anatomical structure of V1 may be used to explore the neural sites of attentional rhythms. Our team provided the first empirical evidence exploring the neural substrate of attentional rhythms by leveraging the anatomical characteristics of V1 (Chen et al. 2018). We presented cues and targets separately to the left and right eye. We discovered identical frequencies of behavioral oscillations under both same‐eye and different‐eye presentation conditions, suggesting that the neural node of attentional rhythms resides in the binocular pathway. However, this study inadvertently included a potential confounding variable: in addition to the inter‐ocular conditions, we also used different visual field presentations. Specifically, the experiment displayed four stimuli on the screen: left visual field stimulus for the left eye, right visual field stimulus for the left eye, left visual field stimulus for the right eye, and right visual field stimulus for the right eye (as illustrated in fig. 2 of Chen et al. 2018). Cues and targets were presented on these four stimuli in each trial. This design necessitated attention to oscillate not only between the left and right eye (inter‐ocular oscillation) but also to switch between the left and right visual field (visual field oscillation). The simultaneous occurrence of inter‐ocular and visual‐field oscillations introduced a potential confound, as their mutual interference—particularly the impact of visual field oscillation on inter‐ocular oscillation—could not be fully excluded. To eliminate the confounding influence of visual field oscillation on attentional rhythms, the present study refines our previous methodology. We now present only one central visual field stimulus to each eye, effectively isolating inter‐ocular oscillation, enabling us to more accurately investigate the neural site underlying attentional rhythms.

Recently, in the study of attentional rhythms system identification, researchers have begun employing a more economical and effective technique has studied (Jia, Fang, and Luo 2019; Jia et al. 2017; VanRullen and MacDonald 2012). This method utilizes EEG with so called Temporal Response Function (TRF) analysis, enabling high‐precision tracking of neural responses to multiple stimuli simultaneously (Crosse et al. 2016; Lalor et al. 2006). Conceptually, the TRF can be likened to an impulse response in engineering systems, representing the brain's (system's) response patterns to specific inputs (stimuli) as a function of time. Similar to event‐related potentials (ERP), the TRF captures time‐locked neural activity but offers additional benefits. For instance, Lalor et al. (2006) demonstrated that the TRF not only achieves exceptionally high temporal resolution but also successfully reconstructs classic ERP components such as the C1, P1, and N1. This makes the TRF a robust tool for studying attentional processes with unparalleled precision and flexibility. More importantly, by applying distinct temporal variation sequences to different stimuli, we can simultaneously track multiple stimuli, achieving a functionality similar to that of the SSVEP technique. In other words, identifying the Impulse Response Function (TRF) combines the advantages of both EEG and SSVEP techniques. Jia et al. (2017) found that in the classic cue‐target paradigm, using this method to calculate the TRFs for left‐side and right‐side stimuli revealed that their activation intensities alternated in a periodic manner (i.e., an anti‐phase pattern), with a frequency consistent with behavioral oscillation frequency. This demonstrated that the TRF's spectrum is an effective measure of attentional oscillations (Jia et al. 2017).

Identifying the Temporal Response Function (TRF) by fitting a filter function is a powerful system identification technique. To obtain a TRF, researchers record electroencephalogram (EEG) data while presenting continuously varying stimuli (such as a sequence of visual stimuli with rapidly and randomly changing brightness, contrast, or motion direction, or the sound envelope of natural speech). In this process, the varying stimulus features are considered as the system's input, while the recorded EEG data are viewed as the system's output. The TRF method assumes that the neural response can be modeled as a linear time‐invariant (LTI) transformation of the stimulus features. By applying regularized linear regression analysis (such as Ridge regression) to the input and output data, the linear mapping relationship between a stimulus feature and its brain response, characterized by the TRF, can be estimated. This process effectively deconvolves the EEG response to obtain the system's impulse response function. The resulting TRF describes how the brain processes specific stimulus features at different time lags, allowing us to study the dynamic processing of continuous natural stimuli by the brain. A TRF can be applied in both forward (stimulus to response) and backward (response to stimulus) directions and can handle multivariate data simultaneously, making its assessment a powerful tool for studying speech processing, attention, audiovisual integration, and other fields. However, it should be noted that the TRF method is based on linear assumptions and may not fully capture nonlinear neural responses. Nevertheless, the TRF estimation provides a robust and flexible approach for studying neural processing under continuous natural stimuli, enabling us to better understand the brain's perceptual processes in everyday life.

This study intends to use impulse response tracking technology to calculate the TRFs for stimuli presented to the left eye and right eye. By observing whether there exists an anti‐phase pattern between the two, we aim to explore whether attention oscillates back and forth between the two eyes, and to determine the role of V1 in attentional oscillations. Based on our work (Chen et al. 2018), in Experiment 1, we presented stimuli in the central visual field of each eye separately to eliminate the confounding effects of oscillations between visual fields and simultaneously used the EEG to obtain the two TRFs of the brain when processing monocular stimuli projected into the visual field of each eye. If the neural sites that generate attentional rhythms are located in the monocular visual pathway, a significant difference between the TRFs and their spectra under the cued‐eye condition and uncued‐eye condition and an obvious antiphase pattern of neural activity should be observed. In contrast, if the neural sites are located in the binocular visual pathway, the theta band and alpha band of TRFs under the two conditions should be in the same phase, and there should be no significant difference. To further confirm that the phase result of the TRFs between the two eyes in Experiment 1 was caused by attentional oscillations between the visual fields of the two eyes, we conducted a control experiment (Experiment 2). We made the attentional system oscillate synchronously in the two eyes by cuing both eyes simultaneously to eliminate visual field oscillations and interocular oscillations. Thus, we sought to obtain two TRFs when the attentional rhythms in both eyes were exactly in the same phase to verify the result from Experiment 1.

## 2 Experiment 1: Attentional Rhythms Under the Monocular Cueing Condition

### 2.1 Methods

#### 2.1.1 Participants

Ten participants (8 females and 2 males, all right‐handed) were recruited for Experiment 1 and provided written consent. The participants were aged 19 to 23 years (*M* = 21.1 years, SD = 1.45 years). All participants had normal or corrected‐to‐normal visual acuity, no color blindness, and no color weakness. The participants were paid after the experiment. This study was conducted in accordance with the Declaration of Helsinki and was approved by the ethical committee of Suzhou University of Science and Technology.

#### 2.1.2 Design

Single‐factor two‐level within‐subject design was used to investigate the interactive patterns of attentional rhythms in the left and right eye of the participants. According to the blinking‐spotlight theory of visual attention, the attentional system always rhythmically oscillates, which enables attention to discretely process a single object and periodically switch between multiple objects (VanRullen 2013, 2016, 2018; VanRullen, Carlson, and Cavanagh 2007). The phases of attentional rhythms are random and disorganized at different time points in each individual. To compare the phases of the TRF oscillations of the left and right eyes, we presented cue stimuli in the visual field of one eye to reset the chaotic phases of attentional rhythms; this manipulation is similar to that used in previous studies (Fiebelkorn, Saalmann, and Kastner 2013; Jia, Fang, and Luo 2019; Jia et al. 2017; Landau and Fries 2012). Then, we observed and measured the phase relationship between the TRF oscillations in the cued‐eye and the uncued‐eye. If an obvious antiphase relationship was present, the attentional system should be able to oscillate between the two eyes rather than only object(s). Pertinently, the neural sites of attentional rhythms are then located in monocular visual pathway. Otherwise, attentional rhythms originate from neural sites in the binocular visual pathway.

To obtain the two TRFs, one for the cued‐eye and one for the uncued‐eye, we used two independent, that is, uncorrelated, random sequences to control the contrast of each disc presented in the visual field of each eye (Jia et al. 2017). Since the two stimulation sequences are statistically independent, the TRFs and their spectra for the two experimental conditions, cued and uncued, can be obtained simultaneously. Importantly, the TRF represents a left‐eye‐specific or right‐eye‐specific response and does not depend on the presence or absence of the target. To maintain the participant's attention state in the experiment, the target was presented in only 25% of the trials.

#### 2.1.3 Apparatus

The procedures were written in MATLAB using the Psychophysics Toolbox‐3 (Brainard 1997; Pelli 1997). The experiment was run on a Dell XPS8700 computer equipped with a GTX1050Ti graphics card. The display was a 22‐in. ViewSonic P225f CRT with a resolution of 1024 × 768 and a refresh rate of 60 Hz. A keyboard was used to document the responses. After linear correction of the screen, a Bits# device was used to generate the stimuli, to ensure the temporal precision of the presentation of the visual stimuli. Furthermore, we adopted Bits# to send triggers to the NeuroScan system to ensure the synchronization precision of the EEG signal and stimulus presentation. Stereoscopes were used to separately reflect images from the left and right side of the computer screen onto the participants' respective eye.

#### 2.1.4 Stimuli

The stimuli were two discs which were black and white radial checkboard patterns and the outer diameter of the two‐disc stimuli was 8°. All the stimuli were presented on a gray (7.53 cd/m^2^) background. Using stereoscopes, the left and right images in the central visual field of both eyes were fused, and the participants could see only the discs. The participants were required to focus on the fixation cross (horizontal and vertical 1° visual angle), which was in the center of the disc stimulus. To improve the fusion of the left and right images, we presented one high‐contrast black and white ring (width: 0.67° visual angle) at 2° visual angle away from the outside of the discs, and 12° further high‐contrast black and white rings (width: 0.17° visual angle) in the center of the discs. We generated two independent temporal sequences from white noise spectra using inverse FFT. The Michelson contrast of the two discs was continuously modulated by these sequences within a range of 0 to 1, synchronized with the screen refresh rate, creating spread spectrum stimuli (Crosse et al. 2016). The luminance of the screen was set to span from 0.01 cd/m^2^ (black) to 67.96 cd/m^2^ (white). The cue was a white ring (width: 1° visual angle) that was presented in the cued‐eye for 500 ms. The target event was a contrast decrement of 500‐ms duration. The contrast of the target was determined by a staircase procedure so that the task difficulty of the experiment could be set at a 79.4% threshold. The target appeared randomly on a certain area of some disc but avoided overlapping with the central fixation point.

#### 2.1.5 Procedures

**FIGURE 1:**
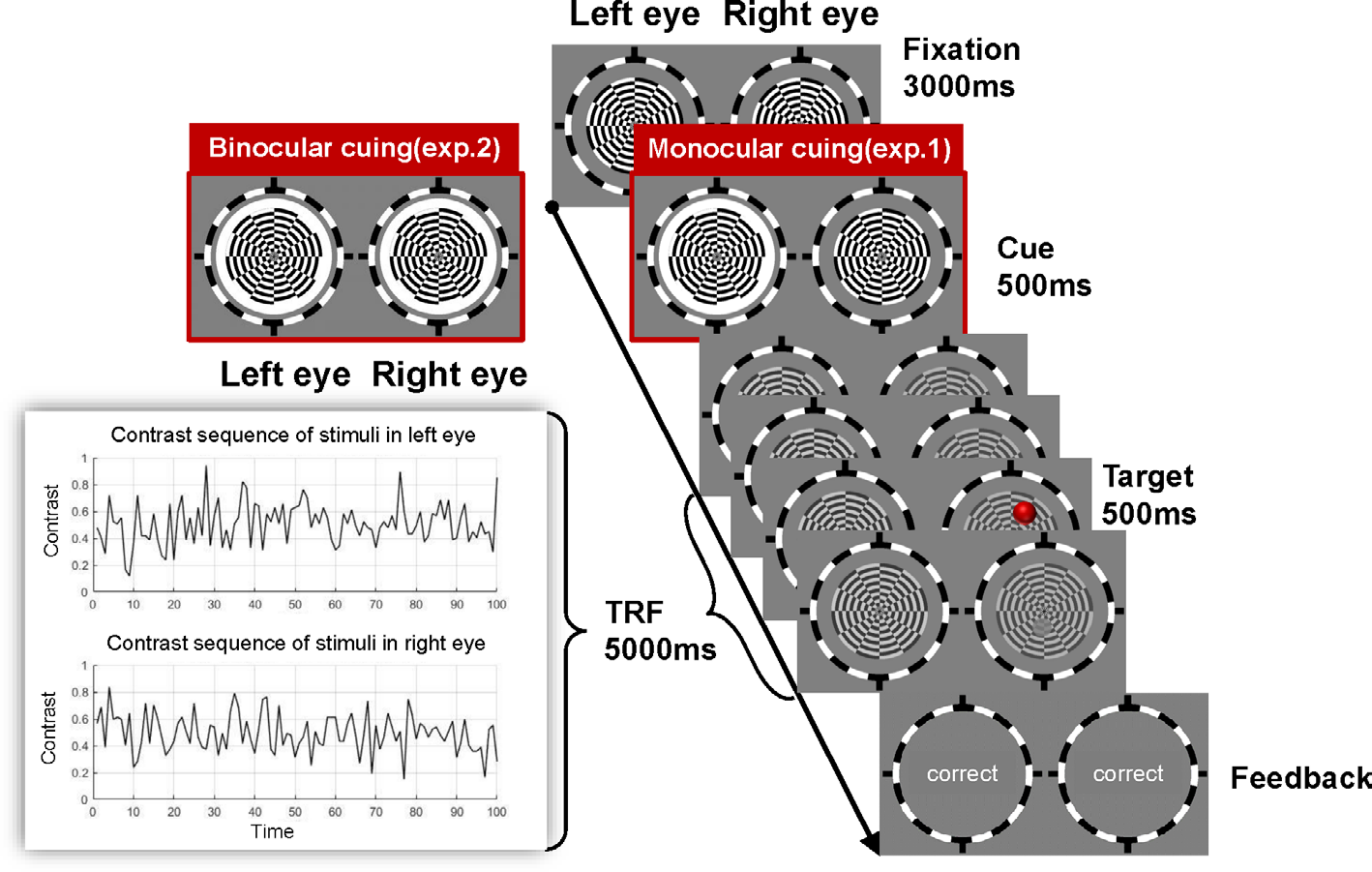
The procedures of Experiment 1 and Experiment 2. The red target in the figure is only for illustration. In the experiment, the target was an unobvious transparency change in a circular area.

The experiment was conducted in a dark room. The participants sat in front of a computer 68 cm away from the screen, with their heads stabilized on a chin rest. Each participant was allowed to adjust the stereoscope until the images projected into the visual fields of the left and right eye were fused well. In each trial, the static disc stimuli were presented for 3000 ms, and a white circle with a width of 1° appeared around the disc stimulus as the cue in a single eye (see Figure 1). After the cue disappeared, the two discs began to flicker and continued to flicker for 5000 ms. In 25% of the trials, while the disc flickered, the target appeared at a random time point between 250 and 4250 ms in the visual field of either the cued‐eye or the uncued‐eye. After the discs disappeared, a white square (with a side length of 1°) appeared at the fixation point, and the participants were asked to press a key then participants needed to determine whether a target was present in each trial. If the target was present, the participants pressed the left arrow on the keyboard. Otherwise, the participants pressed the right arrow on the keyboard. After their responses were collected, after each trial, feedback was presented on the screen, that is, correct, incorrect, or no response. The participants completed the tasks as accurately as possible. There were 128 trials distributed across 7 sessions, with 20 trials in each of the first 6 sessions and 8 trials in the last session. The participants could rest after each session.

### 2.2 Data Analysis

#### 2.2.1 EEG Recording and Preprocessing

EEG data were collected using Synamps 2 amplifiers equipped with a 10–20 standard extended 64‐channel ActiCap (Neuroscan). The sampling rate was 1000 Hz. The data were grounded with the ground electrode placed at the forehead and referenced to location Fz. Vertical electrooculography (EOG) signals were recorded by two electrodes placed above and below the left eye, and the horizontal EOG signals were recorded by another two electrodes placed around the left and right eye. During the experiment, the impedance of the electrodes was kept below 5 kΩ.

Based on previous studies (Jia, Fang, and Luo 2019; Jia et al. 2017), we used the FieldTrip toolbox to process the EEG data. The trials were re‐referenced to the average of all electrodes, bandpass filtered from 2 to 50 Hz, and then epoched from 0 to 5000 ms alignment to the onset of the disc flickering, using a 500 ms prestimulus baseline correction. To avoid the influence of stimulus onset and offset on the TRF, we removed the first 500 ms and the last 1000 ms of EEG data and stimulus contrast sequences from each trial, leaving 3.5 s of data. We then used zero‐padding to supplement the data. Before the TRF was computed, the EEG data and sequences of contrast were spliced together sequentially and converted into z scores.

#### 2.2.2 Calculation of TRFs

We calculated the TRFs using two contrast sequences of stimuli and the corresponding EEG data. The convolution function between the sequence and the corresponding EEG response is *R*(*t*) = TRF*S(*t*). *R*(*t*) represents the neural response, *S*(*t*) represents the stimulus input, and * represents the convolution operation. According to this formula, the disc contrast sequence and EEG data are deconvolved to calculate the TRF of the visual system in response to the disc stimulus contrast. To prevent overfitting in the Ridge regression model, we used a regularization parameter. Then, we calculated the cued‐eye and uncued‐eye TRF for each electrode and each participant. Next, according to previous studies and the topographic map of the EEG data, the data from electrodes POz and Oz were selected for further analysis.

#### 2.2.3 Frequency and Phase Analysis

Fast Fourier transforms (FFT) were used to obtain the spectra of the TRFs of the cued‐eye and uncued‐eye for each participant. To assess the statistical significance of power peaks in the spectrum, we employed a non‐parametric statistical test based on temporal permutations (Landau and Fries 2012; Maris and Oostenveld 2007). This approach assumes that under the null hypothesis of no temporal structure, the time points are exchangeable. The test makes no assumptions about the underlying distribution while accounting for cross‐subject variance (Chen, Wang, et al. 2017; Chen et al. 2020; Landau and Fries 2012). After calculating the TRF, the values of the original TRF were randomly shuffled to disrupt the mapping between the original TRF and its time points, creating randomized data for comparison. Subsequently, an FFT analysis was applied to evaluate the energy contributions potentially arising from random factors across the frequency spectrum of the TRF. To achieve this, we performed 1000 random shuffles to generate pseudosignals, obtaining the amplitude at each frequency for each permutation. These amplitudes formed the permutation distribution for each frequency. By comparing the original energy at each frequency with the permutation distribution, significant oscillation patterns were identified. To account for multiple comparisons in the permutation test, the false discovery rate (FDR) method was employed for correction.

### 2.3 Results

**FIGURE 2:**
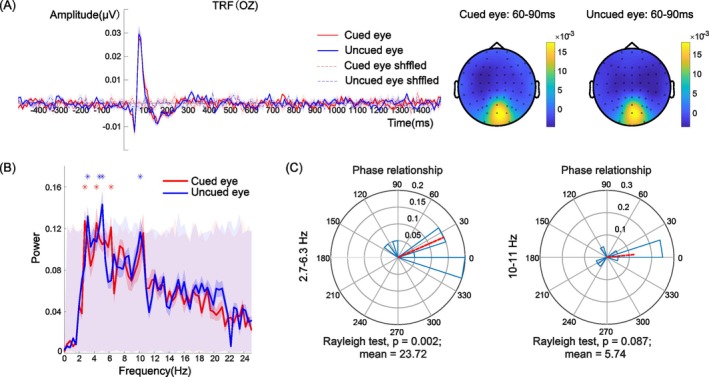
(A) TRF responses and topographic map under the cued‐eye and uncued‐eye conditions at electrode Oz. (B) Power spectrum of the TRF waveform under the cued‐eye and uncued‐eye conditions. The asterisk represents significant frequency points after the permutation test under each condition. The gray shaded area with red low contrast represents the frequency range of 1000 pseudo‐TRFs. (C) Phase relationship between the cued‐eye and uncued‐eye conditions within the theta band (2.76–6.3 Hz) and alpha band (10–11 Hz) neural activity.

**FIGURE 3:**
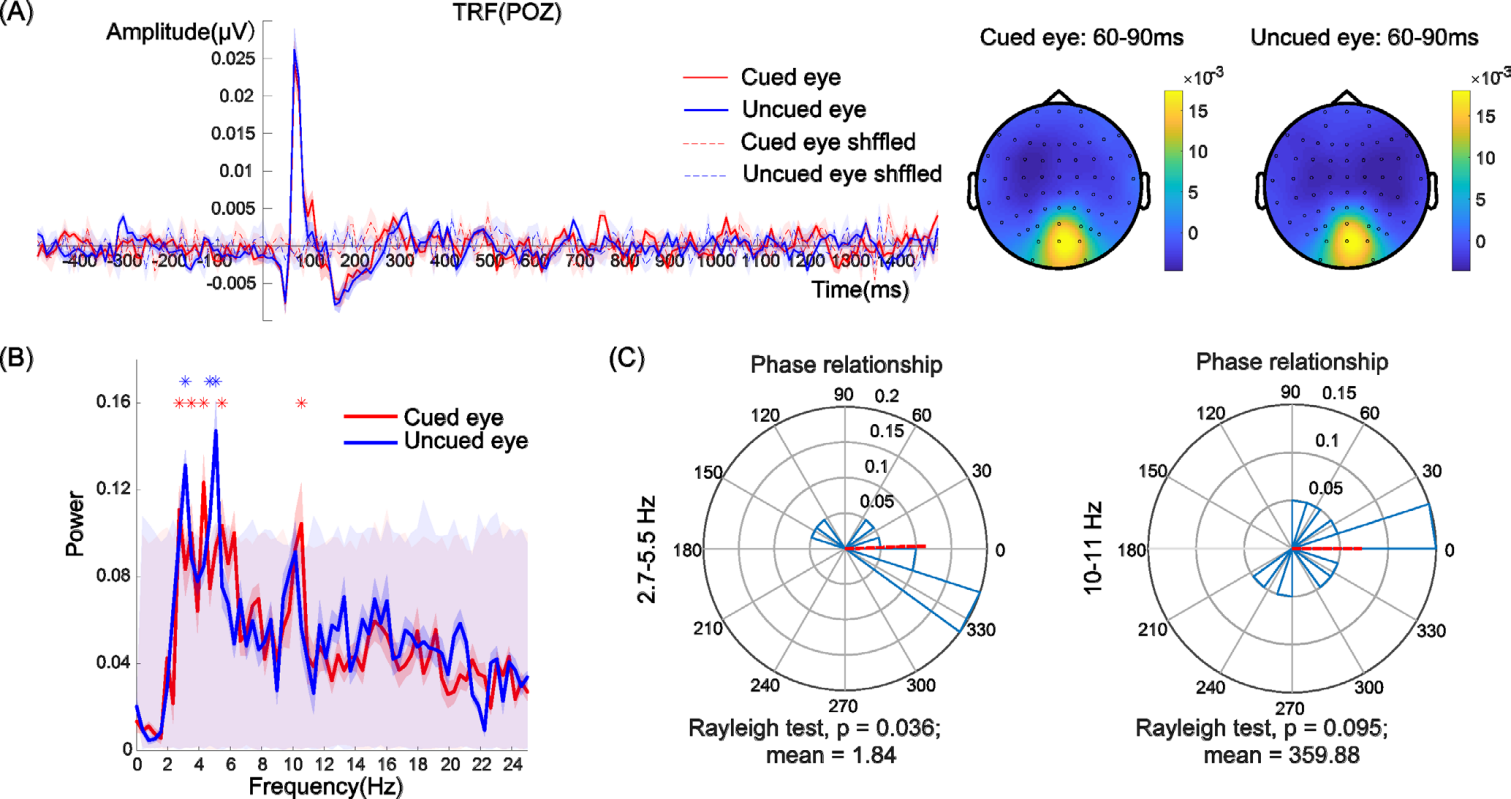
(A) TRF under the cued‐eye and uncued‐eye conditions at electrode POz. Topographic distribution of the TRF amplitude (μV) averaged across the 60–90 ms time window for the cued‐eye (left) and uncued‐eye (right) conditions. The maps reflect the spatial distribution of the TRF response, with warmer colors indicating higher amplitudes and cooler colors indicating lower amplitudes. (B) Spectrum of the TRF under the cued‐eye and uncued‐eye conditions. The asterisk represents significant frequency points after the permutation test under each condition. The gray shaded area with red low contrast represents the frequency range of 1000 pseudo‐TRFs. (C) Phase relationship between the cued‐eye and uncued‐eye conditions with 2.7–5.5 Hz (theta band) and 10–11 Hz (alpha band) neural activity.

The Oz and POz electrodes are close to the early visual cortex and are usually selected for analysis in the TRF approach (Lalor et al. 2006). Therefore, we selected these two electrodes for the frequency and phase analysis. Clear, stable, and effective TRF components are prerequisites for investigating attentional rhythms. To determine the reliability of the TRF response, we shuffled the temporal sequence of the contrast, that is, the convolution relationship in the linear system was shuffled, and the pseudo‐TRFs induced by random factors were obtained (see the dotted line in Figures 2A and 3A). Compared with the pseudo‐TRFs, the TRF response contains prominent C1, P1, and N1 components, which is consistent with the findings of previous studies (Jia et al. 2017; Lalor et al. 2006). This finding suggests that EEG recordings in combination with TRF approach can be used in further analysis.

#### 2.3.1 Oz Electrode

Results for electrode Oz are shown in Figure 2. The power spectra in Figure 2B show strong activation in both the theta and alpha band, for both the cued‐eye and uncued‐eye condition, as evidenced by the power peaks (asterisks denote *p* < 0.05, FDR corrected). The findings regarding these two bands are consistent with the results of behavioral oscillation research (Dugué, Marque, and VanRullen 2015; Fiebelkorn, Saalmann, and Kastner 2013; Landau and Fries 2012). This finding suggests that attentional rhythms were prominent under the both cued‐eye and uncued‐eye conditions (see Figure 2B). To compare the phase differences in the TRFs under the cued‐eye and uncued‐eye conditions, we selected two wide ranges of significant frequencies (2.7–6.3 Hz and 10–11 Hz) for the two TRF spectra in Figure 2B. Figure 2C shows the phase relationship by the phase analysis. The phase difference in the 2.7–6.3 Hz frequency band between the TRF spectra under the cued‐eye and the uncued‐eye conditions was 23.72° (Rayleigh test for circular uniformity, *Z* = 5.64, *p* = 0.002), which showed no significant difference from 0° (i.e., in‐phase mode) and significantly differed from 180° (i.e., antiphase mode; *μ* = 23.72°, 95% CI = [−7.61°, 55.06°]) (Figure 2C left). The phase difference in the 10–11 Hz frequency band between the TRF spectra under the cued‐eye and the uncued‐eye conditions was 5.74° (Rayleigh test, *Z* = 2.42, *p* = 0.087). These two findings suggest that the two TRF oscillations show significant oscillation patterns; however, the two TRFs under the cued‐eye and the uncued‐eye conditions show an in‐phase mode. Notably, we also selected a narrower range (such as 3.1–5.1 Hz) and performed a point‐by‐point phase analysis, and found the same phase relationship using these two analyses as at the wider‐range analysis.

#### 2.3.2 POz Electrode

Results for POz are shown in Figure 3. The TRF responses showed robust theta band (2.7–5.5 Hz) activation and alpha band (10–11 Hz) activation (under the cued‐eye condition) and theta band (3.1–5.1 Hz) activation (under the uncued‐eye condition) (*p* < 0.05, FDR corrected). This finding suggests that attentional rhythms were also prominent at this other occipital electrode under the cued‐eye and uncued‐eye conditions (see Figure 3B). Figure 3C shows the phase relationship. The phase difference in the 2.7–5.5 Hz frequency band between the cued‐eye and uncued‐eye conditions was 1.84° (Rayleigh test, *Z* = 3.23, *p* = 0.036), and showed no significant difference from 0° (i.e., in‐phase mode) and significantly differed from 180° (i.e., antiphase mode; *μ* = 1.84°, 95% CI = [−46.18°, 49.85°]). The phase difference in the 10–11 Hz frequency band between the TRF spectra for the cued‐eye and the uncued‐eye was −0.12° (Rayleigh test for circular uniformity, *Z* = 2.33, *p* = 0.095). This outcome suggests that the theta band and alpha band of the two TRF spectra show significant oscillation patterns; however, the oscillations under the cued‐eye and the uncued‐eye conditions show an in‐phase mode instead of an antiphase mode.

By combining the frequency and phase results of the TRFs at the Oz and POz electrodes, resetting the phase of attentional rhythms in a single eye was not observed to induce the antiphase mode of the TRFs in two eyes; however, a stable in‐phase mode was observed. This finding suggests that the attentional system does not oscillate back and forth between the left and right eye. In contrast, a synchronized oscillation mode between the two eyes is evident, indicating that attentional rhythms originate from the binocular visual pathway of V1 and the higher visual cortex rather than from a monocular visual pathway, which is consistent with our previous behavioral oscillation research (Chen et al. 2018). Notably, although the in‐phase oscillation mode induced by monocular cues can rule out the possibility that a monocular visual pathway produces attentional rhythms, whether the TRFs produced by a monocular cue (Experiment 1) are the same as the TRFs when binocular cells are activated is still unknown.

## 3 Experiment 2: Attentional Rhythms Under Binocular Cuing Conditions

To further investigate the credibility of the TRF in‐phase oscillation in Experiment 1, in Experiment 2 the cue was presented in the left eye and right eye simultaneously to reset the phases of the attentional rhythms in both eyes; this artificially made the attention system oscillate synchronously between the two eyes. If the interaction mode of the TRFs in both eyes observed in Experiment 2 is the same as that in Experiment 1, the results in Experiment 1 are suggested to be caused by “attentional synchronization oscillation,” thus indicating that attentional rhythms originate from the binocular visual pathway, which can be activated by both monocular cues (Experiment 1) and a binocular cue (Experiment 2).

### 3.1 Methods

Except for the cue stimulus in Experiment 2 being presented in both eyes simultaneously (see Figure 1), the other stimuli and analysis methods were the same as those in Experiment 1.

### 3.2 Results

**FIGURE 4:**
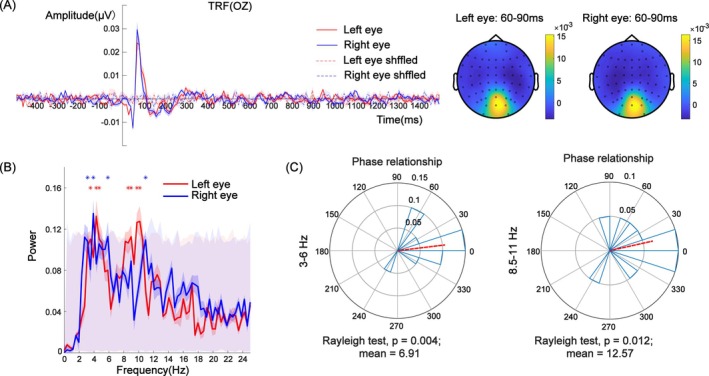
(A) TRF and topographic map of the two eyes at electrode Oz. Topographic distribution of the TRF amplitude (μV) averaged across the 60–90 ms time window for the cued‐eye (left) and uncued‐eye (right) conditions. The maps reflect the spatial distribution of the TRF response, with warmer colors indicating higher amplitudes and cooler colors indicating lower amplitudes. (B) Power spectrum of the TRF waveform of the two eyes. The asterisk represents significant frequency points after the permutation test under each condition. The gray shaded area with red low contrast represents the frequency range of 1000 pseudo‐TRFs. (C) Phase relationship under 3–6 Hz (theta band) and 8.5–11 Hz (alpha band) neural activity.

**FIGURE 5:**
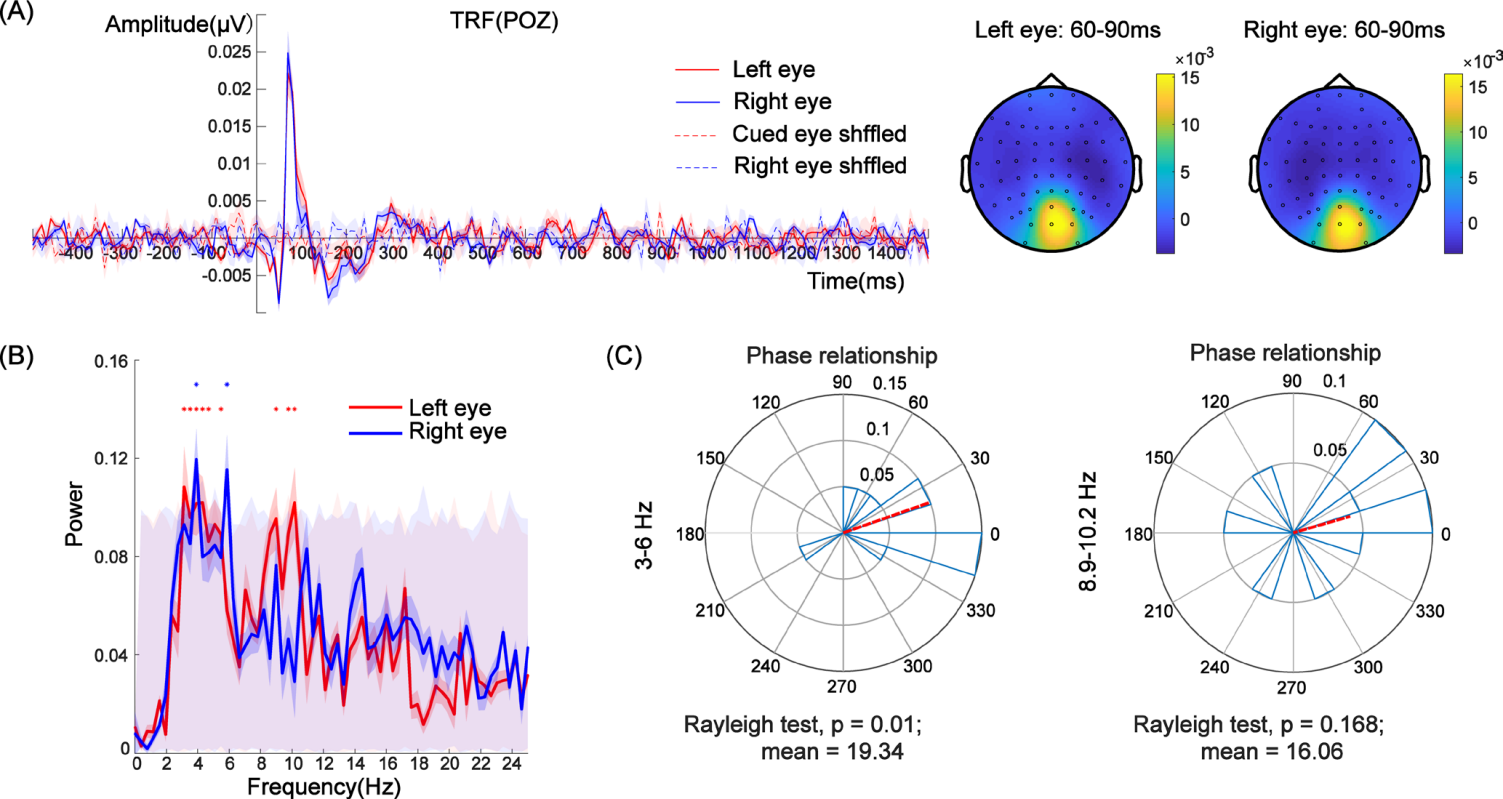
(A) TRF responses and topographic map of the two eyes at electrode POz. Topographic distribution of the TRF amplitude (μV) averaged across the 60–90 ms time window for the cued‐eye (left) and uncued‐eye (right) conditions. The maps reflect the spatial distribution of the TRF response, with warmer colors indicating higher amplitudes and cooler colors indicating lower amplitudes. (B) Power spectrum of the TRF waveform of the two eyes. The asterisk represents significant frequency points after the permutation test under each condition. The gray shaded area with red low contrast represents the frequency range of 1000 pseudo‐TRFs. (C) Phase relationship with 3–6 Hz (theta band) and 8.9–10.2 Hz (alpha band) neural activity.

We explored the interaction mode of the TRF responses between the left eye and right eye in Experiment 2. As before, in order to obtain a measure of reliability of the TRF, the temporal sequences of the stimulus contrast were shuffled to obtain the pseudo‐TRF induced by random factors (see Figure 4A and the dashed part). Compared with the pseudo‐TRF, the TRF signal in Experiment 2 contained prominent C1, P1, and N1 components (Figures 4A and 5A), which is consistent with previous studies (Jia et al. 2017; Lalor et al. 2006), thus indicating that the TRF in this experiment is highly reliable. Therefore, the TRF, viz. its spectrum, can be used as a physiological indicator of attentional rhythms in further analysis. As before we analyzed, in particular, the data at the two occipital electrodes, Oz and POz.

#### 3.2.1 Oz Electrode

Results for electrode Oz are shown in Figure 4. The TRF's power spectrum shown in Figure 4B showed strong theta band (3.5–4.7 Hz) and alpha band (8.5–10.2 Hz) activations in the left eye and theta band (3–6 Hz) and alpha band (10–11 Hz) activation in the right eye (*p* < 0.05, FDR corrected) as evidenced by the power peaks in the respective frequency ranges. The findings of these two bands are consistent with the results in Experiment 1. This finding suggests that attentional rhythms were present under each eye condition (see Figure 4B). To compare the phase differences in the TRFs of the two eyes, we conducted a phase analysis in two ranges of frequencies (see Figure 4C). The phase difference in the 3–6 Hz frequency band in the TRF responses between the two eyes was 6.91° (Rayleigh test, *Z* = 4.98, *p* = 0.004), which showed no significant difference from 0° (i.e., in‐phase mode) and significantly differed from 180° (i.e., antiphase mode; *μ* = 6.91°, 95% CI = [−27.59°, 41.41°]). The phase difference in the 8.5–11 Hz frequency band in the TRFs was 12.57° (Rayleigh test, *Z* = 4.14, *p* = 0.012), which showed no significant difference from 0° (i.e., in‐phase mode) and significantly differed from 180° (i.e., antiphase mode; *μ* = 12.57°, 95% CI = [−27.08°, 52.21°]). These findings suggest that the two TRFs showed significant, pronounced oscillation patterns. More importantly, the TRF responses showed an in‐phase mode instead of an antiphase mode.

#### 3.2.2 POz Electrode

We analyzed the neural activity recorded at electrode POz. The TRF's power spectrum shown in Figure 5B showed strong theta band (3–5.5 Hz) and alpha band (8.9–10.2 Hz) activations in the left eye and theta band (3.9–6 Hz) activation in the right eye (*p* < 0.05, FDR corrected) as evidenced by the power peaks in the respective frequency ranges. The findings of these two bands are consistent with the results of Experiment 1. This finding suggests that attentional rhythms were obvious under each eye condition (see Figure 5B). To compare the phase differences in the TRFs between the two eyes, we conducted a phase analysis in two ranges of frequencies (see Figure 5C). The phase difference in the 3–6 Hz frequency band in the TRF responses between the two eyes was 19.34° (Rayleigh test, *Z* = 4.28, *p* = 0.01), which showed no significant differences from 0° (i.e., in‐phase mode) and significantly differed from 180° (i.e., antiphase mode; *μ* = 19.34°, 95% CI = [−19.29°, 57.98°]). In addition, the neural activity of the 8.9–10.2 Hz frequency band showed no significant phase relationship (Rayleigh test, *Z* = 1.80, *p* = 0.17). This finding suggests that the two TRFs show significant oscillation patterns with an in‐phase relationship instead of an antiphase relationship.

By combining the frequency and phase results of the TRFs' spectra at the Oz and POz electrodes, presenting cues in two eyes simultaneously can be shown to induce obvious attentional rhythms. Specifically, there was an in‐phase mode between the two TRF responses, further supporting that the in‐phase oscillation patterns observed in Experiment 1 indeed arise from the neural activity of the binocular visual pathway, which is consistent with our hypothesis. A one‐way analysis of variance (ANOVA) was used to compare the phase differences between Experiment 1 and Experiment 2. We found that at electrode Oz, there were no significant phase differences in the band range of 2.7–6.3 Hz, *F*(1, 18) = 0.47, *p* = 0.50, and the band range of 8.5–11 Hz, *F*(1, 18) = 0.33, *p* = 0.58; in addition, at electrode POz, there were no significant phase differences in the band range of 2.7–6.3 Hz, *F*(1, 18) = 0.10, *p* = 0.76, and in the band range of 8.5–11 Hz, *F*(1,18) = 1.31, *p* = 0.27. These findings further support the results and conclusions of Experiment 1.

## 4 General Discussion

In the current study, we employed EEG recordings in conjunction with a temporal response function (TRF) approach (i.e., extracting the impulse response function of a sensory system; Crosse et al. 2016) to examine the cortical neural activities generated by the left and right eye. First, we found that attentional rhythms manifested themselves in the alpha and theta bands in the two experiments. Second, regardless of whether the cues were presented in only one eye (Experiment 1) or both eyes (Experiment 2), the interaction pattern between the TRFs of each eye showed no significant difference and was always in phase, indicating it was difficult for the cue stimulus to interfere with attentional rhythms in one eye alone. Thus, attention scarcely oscillated between the two eyes, implying that the neural sites that produce attentional rhythms are located in the binocular visual pathway.

Our results provide important neural evidence of attentional rhythms consistent with our previous behavioral oscillation research (Chen et al. 2018), further confirming the role of the binocular visual pathway in attentional rhythms. In a previous study, we used a high‐resolution cue‐target paradigm and found that, regardless of whether the cue and the target were presented in the same eye or different eyes, the prominent frequencies of behavioral oscillations were the same, proving that attentional oscillations originate in the binocular visual pathway (Chen et al. 2018).

Moreover, our results imply that the neural sites of attentional rhythms are located in the binocular visual pathway, which is consistent with the findings of previous studies. The phases of the theta band on occipital electrodes recorded with intracranial electrodes in epilepsy patients predict fluctuations in accuracy and reaction time (Helfrich et al. 2018). The theta band of neural oscillations in awake monkeys' V4 cortex has also been shown to be the neural basis of 4 Hz behavioral oscillations (Kienitz et al. 2018). Relevantly, theta oscillations and theta band synchronization in the V1–V2 and V4‐TEO regions are closely related to attentional rhythms (Spyropoulos, Bosman, and Fries 2018). Cortical distance modulates attentional rhythms, and this modulation may be attributed to early cortical areas, such as V1, V2, and V3 (Chen et al. 2020). Using a TRF approach, neural oscillations in the central posterior parietal lobe have been shown to be significantly related to behavioral oscillations (Jia, Fang, and Luo 2019). Notably, while monkeys performed an object‐based attention task, Fiebelkorn et al. recorded their local field potentials and found that the phase of the theta band on the top frontal eye field (FEF) and lateral intraparietal area (LIP) can predict the performance of behavioral oscillations (Fiebelkorn and Kastner 2019). Finally and importantly, in the above research, the occipital lobe, V4, posterior central parietal lobe, FEF, and LIP were found in the binocular visual pathway, which is consistent with the results of this study.

Our findings provide supporting evidence for the prediction of the Feedforward‐Feedback‐Verify‐reWeight (FFVW) model proposed by Zhaoping (2017, 2021, 2022). In this model, information from the two eyes is fed forward from V1 to higher brain areas. In ambiguous situations, these feedforward signals suggest multiple perceptual hypotheses about the visual scene. To reach a perceptual outcome, higher areas synthesize the would‐be visual inputs for each hypothesis according to their internal models about the visual world, and the would‐be inputs are fed back to V1 to verify whether they match the actual feedforward visual input. The hypothesis with the best match is then given a higher weight to become the perceptual outcome. Importantly, this synthesis‐feedback‐verify process in FFVW is an attentional process, and since our brain has an internal model that visual inputs from the two eyes are highly correlated, the synthesized would‐be inputs are binocular; accordingly, FFVW predicts that the feedback should be directed to binocular neurons in V1 for verification, even though feedforward signals from V1 arise from both the binocular and monocular channels. It should be noted that the feedback process is an important mechanism for top‐down visual attention. Thus, the FFVW prediction that feedback mainly targets the binocular channel implies that top‐down attention is biased for binocular inputs, so that attentional weights to the inputs from the two eyes should be roughly equal. Hence, our findings provide evidence for FFVW, while FFVW provides a theoretical base for our research.

The present study, based on anatomical features of the primary visual cortex, further found that attentional rhythms arise from the binocular visual pathway rather than the monocular visual pathway.

## Ethics Statement

Written informed consent was obtained. This study was conducted in accordance with the Declaration of Helsinki and was approved by the ethical committee of Suzhou University of Science and Technology.

## Conflicts of Interest

The authors declare no conflicts of interest.
